# Lymph node metastatic patterns and its clinical significance for thoracic superficial esophageal squamous cell carcinoma

**DOI:** 10.1186/s13019-020-01302-z

**Published:** 2020-09-21

**Authors:** An Wang, Lu Lu, Jie Fan, Shaohua Wang, Xiaofeng Chen

**Affiliations:** 1 Department of Thoracic Surgery, Huashan Hospital, Fudan University, Shanghai, China; 2 Department of Pathology, Huashan Hospital, Fudan University, Shanghai, China

**Keywords:** Superficial esophageal carcinoma, Lymph node, Squamous cell carcinoma

## Abstract

**Background and objectives:**

The optimal therapeutic method for patients with superficial esophageal squamous cell carcinoma (sESCC) remains to be established.

**Methods:**

Clinical data of all the patients from 2002 to 2014 who underwent curative esophagectomy and three-field lymphadenectomy for thoracic sESCC were collected based on a prospectively-maintained database. The pattern of lymph node metastasis was analyzed based on the depth of tumor invasion, tumor location and surgical fields.

**Results:**

The involved lymph node region was associated to the tumor location, however, upper mediastinal and perigastric region was the most vulnerable region. The incidence of lymph node metastasis increased with the depth of tumor invasion. No lymph node involvement was found in tumors invading proper mucosa (M2), while the pattern of positive lymph nodes in tumors invading the deepest 1/3 submucosa was similar to that in advanced ESCC. Lymphatic invasion, tumor location and upper mediastinal lymph node involvement were independent predictors for cervical lymph node metastasis. For patients without lymphatic invasion, the positive predictive value of upper mediastinal lymph node metastasis for positive cervical lymph node was low (0 ~ 25%), while the negative predictive value was extremely high, wherever the tumor located (93.8 ~ 100%).

**Conclusions:**

Tumors invading till proper mucosa was the best indication for endoscopic mucosa resection. Mediastinal-abdominal lymphadenectomy was essential for sESCC invading beyond proper mucosa. For those without lymphatic invasion, cervical lymphadenectomy might be avoided in case of negative upper mediastinal lymph node.

## Introduction

With the advent and development of multimodality therapy, many patients with superficial esophageal squamous cell carcinoma(sESCC) can benefit from endoscopic mucosa resection for its low possibility of lymph node metastasis [1]. As the most significant prognostic factor for esophageal squamous cell carcinoma [2], the status of lymph node metastasis is the basis for therapy choice. However, currently there is no reliable pre-excision molecular, biological or immunohistochemical predictive markers of lymph node metastasis in superficial esophageal cancer, and the diagnostic performance of preoperative workup for nodal disease is poor [3]. There are literatures [4–6] which demonstrated the prevalence and characteristics of lymph node involvement in superficial esophageal cancer, however the significance of the results is limited because of the heterogeneity in histological type and surgical methods. Herein we summarized the patterns of lymph node metastasis assessed by three-field lymphadenectomy in sESCC in our institute with a period of 12 year, aiming to investigate the best therapeutic recommendations for sESCC.

## Methods

### Patient enrollment

**Fig. 1:**
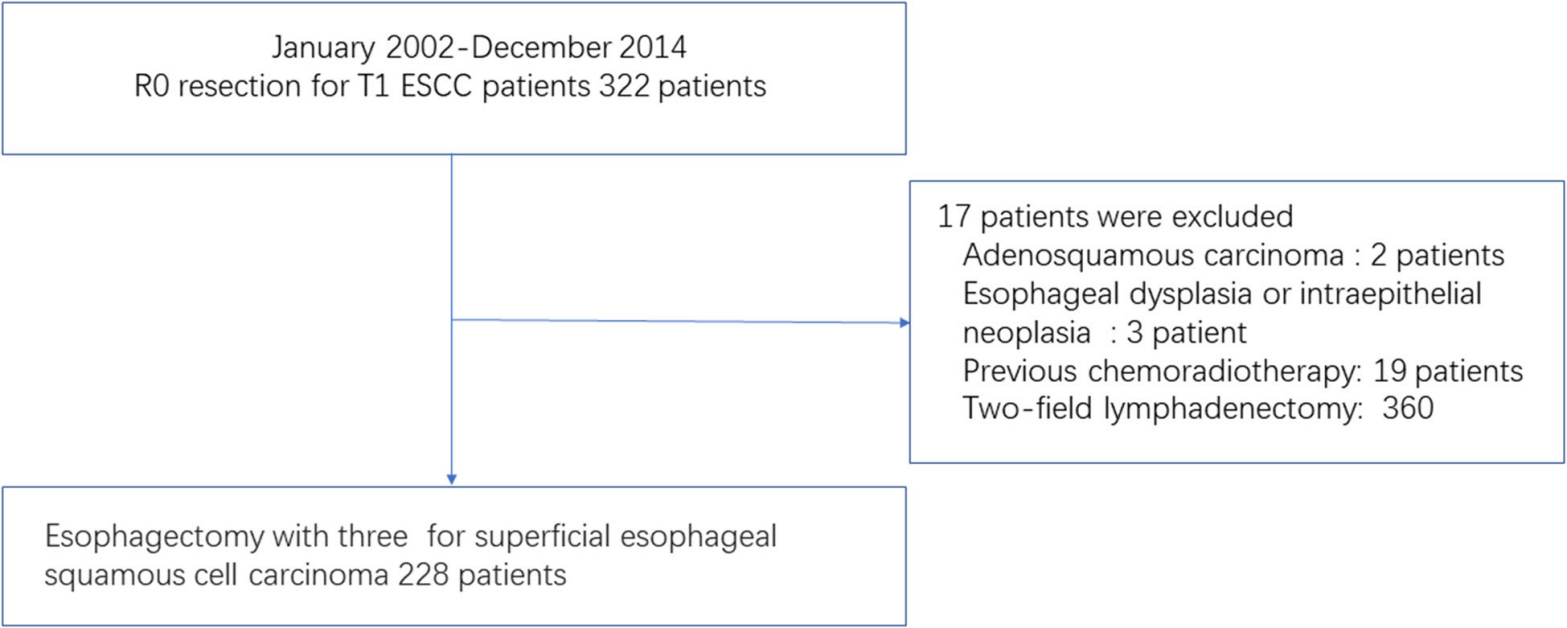
Patient selection process

We conducted computerized and manual searches with the keyword ‘T category = 1’, ‘right transthoracic surgical procedure’, ‘R0 resection’ and ‘histology type = ESCC’ in our prospectively-maintained database; the patients who had received preoperative chemotherapy or radiotherapy were excluded (Fig. 1). At last, there remained 228 patients, who received esophagectomy and cervical-mediastinal-abdominal lymphadenectomy with curative intention for thoracic sESCC from the beginning of 2002 to the end of 2014.

### Evaluation of tumor invasive depth

Recently, the pathological subclassication of the extent of the cancerous invasion in supercial esophageal cancer has been commonly utilized [Endo, Leers]. Depth of tumor invasion was classified as mucocal (m) and submucosal(sm) based on whether there was tumor invasion through the deepest muscular fibers of the muscularis mucosa. Both mucosal cancer and submucosal cancer were each further divided into three subtypes according to the extent of invasion, i.e. m1, m2, m3 cancers and sm1, sm2, sm3 cancers. Cases of m1 cancer included epithelial cancer, including cases in which it was questionable whether or not there was invasion to the lamina propria mucosae. Cancer invasive to the lamina propria mucosae was classied as m2, and m3 cancer cases included cases with invasion in contact with or into the muscularis mucosae. Submucosal cancers were also divided into three subtypes: sm1 cancer consisted of cases with invasion within the upper third layer of the submucosa, invasion to the middle third layer of the submucosa was classified as sm2 and invasion as far as the lower third layer of the submucosa was classified as sm3.

Representative slices of H.E. staining were re-evaluated by two pathologists (a deputy chief physician and an attending physician), who were blinded to surgical oncological results. They made independent assessment on tumor invasive depth using the new naming subclassification system described above. Disagreements were resolved through consensus on a multi-head microscope.

### Data collection

All patient data, including demographic characteristics, tumor location, tumor differentiation, pathological T category, pathological N category, pathological M category, pathological TNM stage and adjuvant therapeutic regime were collected from our prospectively-maintained database. Staging was judged pathologically using the 7th edition of the UICC TNM classification system.

### Surgical procedure for esophagectomy and 3-field lymphadenectomy

The standard surgical procedure used in our study was a combination of esophagectomy with mediastinal lymphadenectomy via right thoracotomy, upper abdominal lymphadenectomy, reconstruction with a gastric tube via the posterior mediastinum, and anastomosis in the cervical incision. 3-FL lymphadenectomy was adopted for all patients. We removed the left recurrent laryngeal nerve lymph nodes from the right thoracic cavity. Cervical lymph nodes contained both sides of the supraclavicular lymph nodes, middle deep cervical lymph nodes and cervical paraesophageal lymph nodes in 3-FL lymphadenectomy.

### Areas of lymph node metastasis

The lymph node metastatic pattern of sESCC was investigated according to the tumor location and tumor invasive depth. The lymph node areas were divided as follows: (1), cervical lymph nodes; (2), upper mediastinal area including paratracheal nodes, nodes along both recurrent laryngeal nerves and upper paraesophageal nodes; (3), mid-mediastinal area including middle paraesophageal nodes and subcarinal nodes; (4), lower mediastinal area from the caudal margin of the inferior pulmonary vein including lower paraesophageal nodes and diaphragmatic nodes; and (5),abdominal area including paragastric nodes, common hepatic nodes, splenic nodes, and celiac nodes.

### Evaluation of lymphovascular invasion

In the present study, lymphovascular invasion was defined as lymphatic and (or) vascular invasion. The method to evaluate and distinguish lymphatic invasion from vascular invasion was described in our previous study [7]. Briefly, the presence of erythrocytes within the involved vessel lumen and the relatively large diameter of the involved vessel helped to distinguish vascular invasion from lymphatic invasion. For difficult cases or those cases without definitively positive evidence of lymphovascular invasion by haematoxylin and eosin (HE) staining, CD34 staining, which detects vascular endothelium, and podoplanin staining, which detects lymphatic endothelium were applied in representative slices for better detection and distinction. The evaluation of lymphovascular invasion was independently performed by two experienced pathologists, both of whom were blinded to the surgical oncological information. Disagreements were discussed and resolved through consensus.

### Statistics analysis

Statistics analysis was conducted using the SPSS 19.0 Statistics Software Package (SPSS Inc., Chicago, IL). Unless stated otherwise, mean values and standard deviations are reported. Student’s *t*-test was used for comparisons between subgroups. For categorical variables, the χ^2^ test or Fisher’s exact test was used as appropriate. For identifying risk factors, those variables with a *p* value less than 0.1 according to univariate analysis were then included into a binary logistics regression analysis using the backward wald stepwise method. For all statistical analyses, *p* values less than 0.05 were considered significant.

## Results

### Demographics and clinicopathologic characteristics of all patients

The demographics and clinicopathological characteristics were listed in Table 1. The surgical mortality was 3/228 (1.3%). The median of removed regional lymph node was 34 (8–70). The median of removed lymph node in cervical, thoracic and abdominal area was 18, 20 and 13 respectively.

**Table 1:** Demographics and clinicopathologic characteristics

Variables	N(%)
Gender	
Male (%)	202 (88.6)
Female (%)	26 (11.4)
Age (y)	median:64, min-max:41–82
Tumor location	
Upper Tx (%)	34 (14.9)
Mid Tx (%)	125 (54.8)
Lower Tx (%)	69 (30.3)
Macroscopic tumor type	
0-I	70 (30.7)
0-IIa	45 (19.7)
0-IIb	3 (1.3)
0-IIc	103 (45.2)
0-III	7 (3.1)
Tumor length(mm)	median:36, min-max:2–180
Differentiation	
Well (%)	58 (25.4)
Moderate (%)	86 (37.7)
Poor (%)	84 (36.8)
Growth pattern	
Expansive (%)	82 (36.0)
Infiltrative (%)	146 (64.0)
Lymphatic invasion	
Absent (%)	166 (72.8)
Present (%)	62 (27.2)
Vascular invasion	
Absent (%)	189 (82.9)
Present (%)	39 (17.1)
pT category	
T1a (%)	62 (27.2)
M2	27
M3	35
T1b (%)	166 (72.8)
SM1	57
SM2	61
SM3	48
pN category	
N0 (%)	135 (59.2)
N1 (%)	69 (30.3)
N2 (%)	19 (8.3)
N3 (%)	5 (2.2)
pM category	
M0 (%)	212 (93.0)
M1 (lym) (%)	16 (7.0)
pStage	
Stage I(%)	133 (58.3)
Stage II(%)	64 (28.1)
Stage III(%)	15 (6.6)
Stage IV (lym)(%)	16 (7.0)

*Tx* thorax, *NS* no significance

### Prevalence of lymph node metastasis in different areas according to tumor location and depth of tumor invasion

None of the cases with tumor invading mucosa proper lamina (m2) had lymph node metastasis. Generally speaking, the most susceptible lymph nodes were in upper mediastinal area (48/228, 21.1%) and abdominal perigastric area (47/228, 20.6%). Tumors located at upper thoracic esophagus were most likely to have lymph node metastasis at upper mediastinal (15/34, 44.1%) and cervical area (8/34, 23.5%), while it was uncommon for tumors located at lower thoracic esophagus to develop lymph node metastasis at upper mediastinal area (4/69, 5.8%). Few patients with tumor located at lower thoracic esophagus had cervical lymph nodes metastasis (1/69, 1.4%). For tumors invading the deepest 1/3 layer of submucosa, the prevalence of lymph node metastasis in the upper mediastinal area and perigastric area was about 40%. The prevalence of lymph node metastasis increased with the depth of tumor. No matter which layer of submucosal was involved, the most common involved area was upper mediastinum and abdominal perigastric area (Table 2).

**Table 2:** Primary tumor location, tumor invasion depth and areas of nodal metastasis

	Total	Tumor location				Tumor invasive depth				
	*N* = 228 (%)	Upper (*n* = 34)(%)	Mid (*n* = 125)(%)	Lower (*n* = 69)(%)	*P* value	M3 (*n* = 35)(%)	SM1 (*n* = 57)(%)	SM2 (*n* = 61)(%)	SM3 (*n* = 48)(%)	*P* value
Cervical	23 (10.1)	8 (23.5)	14 (11.2)	1 (1.4)	0.002	2 (5.7)	3 (5.3)	6 (9.8)	12 (25.0)	0.002
Upper mediastinum	48 (21.1)	15 (44.1)	29 (23.2)	4 (5.8)	< 0.001	1 (2.9)	12 (21.1)	16 (26.2)	19 (39.6)	< 0.001
Mid-mediastinum	12 (5.3)	2 (5.9)	8 (6.4)	2 (2.9)	NS	1 (2.9)	2 (3.5)	1 (1.6)	8 (16.7)	0.003
Lower mediastinum	21 (9.2)	2 (5.9)	12 (9.6)	7 (10.1)	NS	2 (5.7)	5 (8.8)	4 (6.6)	10 (20.8)	0.02
Perigastric	47 (20.6)	2 (5.9)	26 (20.8)	19 (27.5)	0.038	3 (8.6)	10 (17.5)	14 (23.0)	20 (41.7)	< 0.001

### Operative fields distribution of metastatic lymph nodes according to tumor location

Tumors located at lower thoracic segment (Table 3) never had separate cervical lymph node metastasis while cervical lymph node metastasis occurred in patients with simultaneous thoracic and abdominal lymph node involvement. Few patients whose tumors were at upper thoracic esophagus had abdominal lymph node metastasis, among whom there was 1 patient with separate abdominal lymph node metastasis. Tumors at middle thoracic esophagus tended to spread cephalicly and caudally to all the three operative fields (Table 3).

**Table 3:** Lymph node metastatic characteristics according to operative fields in positive node disease

Number of cases with nodal disease according to operative field (%)	Tumor Location		
	Upper Tx *N* = 19	Middle Tx *N* = 52	Lower Tx *N* = 25
1-field			
Cervical	2 (10.5)	2 (3.8)	0
Thoracic	10 (52.6)	19 (36.5)	6 (24.0)
Abdominal	1 (5.3)	9 (17.3)	15 (60.0)
2-field			
Cervical-Thoracic	5 (26.3)	5 (9.6)	0
Thoracic-Abdominal	0	10 (19.2)	3 (12.0)
Cervical-Abdominal	1 (5.3)	3 (5.8)	0
3-field			
Cervical-Thoracic-Abdominal	0	4 (7.6)	1 (4.0)

*Tx* thoracic

### Analysis of correlated factors for cervical lymph node metastasis

Univariate analysis (Table 4) showed that age (< 65y) (*p* = 0.086), tumor location (*p* = 0.002), lymphatic invasion (*p* <  0.001), depth of tumor invasion (*p* = 0.002), and positive lymph node involvement in other area (upper mediastinal area, *p* <  0.001; middle mediastinal area, *p* = 0.003; lower mediastinal area, *p* = 0.011; abdominal perigastric area, *p* = 0.029) were potentially correlated with cervical lymph node metastasis. Multivariate analysis (Table 4), the efficacy of which was demonstrated by a Chi-square test (*p* <  0.001) demonstrated that tumor location (*p* = 0.006), lymphatic invasion (*p* = 0.001), and upper mediastinal lymph node metastasis (*p* = 0.03) were independent correlated factors with cervical lymph node involvement.

**Table 4:** Univariate and multivariate analysis of predictors for cervical lymph node metastasis in superficial thoracic ESCC

Variables	Univariate analysis			Multivariate analysis		
	Cervical LN(−)(%)	Cervical LN(+) (%)	*P* value	HR	95% CI for HR	*P* value
Age			0.086	–	–	0.283
< 65 y	104 (86.7)	16 (13.3)				
≥ 65 y	10 (93.5)	7 (6.5)				
Gender			0.911	–	–	–
Male	180 (89.1)	22 (10.9)				
Female	25 (96.2)	1 (3.8)				
Tumor location			0.002	0.304	0.130–0.711	0.006
Upper thorax	26 (76.5)	8 (23.5)				
Middle thorax	111 (88.8)	14 (11.2)				
Lower thorax	68 (98.6)	1 (1.4)				
Tumor size			0.888	–	–	–
≤ 4 cm	119 (90.2)	13 (9.8)				
> 4 cm	86 (89.6)	10 (10.4)				
Macroscopic tumor type			0.914	–	–	–
0-II	136 (90.1)	15 (9.9)				
0-I/0-III	69 (89.6)	8 (10.4)				
Tumor differentiation			0.128	–	–	–
Well differentiation	53 (91.4)	5 (8.6)				
Moderate differentiation	73 (84.9)	13 (15.1)				
Poor differentiation	79 (94.0)	5 (6.0)				
Growth pattern			0.134	–	–	–
Expansive	77 (93.9)	5 (6.1)				
Infiltrative	128 (87.7)	18 (12.3)				
Lymphatic invasion			< 0.001	6.454	2.202–18.919	0.001
Absence	159 (95.8)	7 (4.2)				
Presence	46 (74.2)	16 (25.8)				
Vascular invasion			0.560	–	–	
Absence	171 (90.5)	18 (9.5)				
Presence	34 (87.2)	5 (12.8)				
Pathological T category			0.002	–	–	
T1a (%)						
M2	27 (100)	0				
M3	33 (94.3)	2 (5.7)				
T1b (%)						
SM1	54 (94.7)	3 (5.3)				
SM2	55 (90.2)	6 (9.8)				
SM3	36 (75.0)	12 (25.0)				
Upper mediastinal LN metastasis			< 0.001	3.196	1.118–9.136	0.03
Absence	171 (95.0)	9 (5.0)				
Presence	34 (70.8)	5 (29.2)				
Mid mediastinal LN metastasis			0.003			
Absence	198 (91.7)	18 (8.3)				
Presence	7 (58.3)	5 (41.7)				
Lower mediastinal LN metastasis			0.011			
Absence	190 (91.8)	17 (8.2)				
Presence	15 (71.4)	6 (28.6)				
Abdominal mediastinal LN metastasis			0.029			
Absence	167 (92.3)	14 (7.7)				
Presence	38 (80.9)	9 (19.1)				

*LN* lymph node, *CI* confidence interval, *HR* hazard ratio, *ESCC* esophageal squamous cell carcinoma

### Predictive value of the status of upper mediastinal lymph nodes for the status of cervical lymph nodes

Patients were stratified by the status of lymphatic invasion and tumor location (Table 5). For patients with negative lymphatic invasion, the positive predictive value of upper mediastinal lymph node metastasis for positive cervical lymph node was low (0 ~ 25%, depending on the tumor location), while the negative predictive value was extremely high, wherever the tumor located (93.8 ~ 100%). For patients with positive lymphatic invasion, the positive predictive value increased to 42.9 ~ 61.5% for tumors located at upper or middle thoracic esophagus, while the negative predictive value decreased to 33.3% for upper thoracic sESCC. For middle or lower thoracic sECSS, the negative predictive value was decreased but was still high (88.9 ~ 94.1%).

**Table 5:** Analysis of relationship between the status of upper mediastinal lymph nodes and the status of cervical lymph nodes

Lymphatic invasion	Tumor location	Upper mediastinal LN	Cervical LN-	Cervical LN	Positive predictive value (%)	Negative predictive value (%)	Auucracy (%)
			–	+			
–	Upper Tx	–	15	1	25.0	93.8	70.8
		+	6	2			
	Mid Tx	–	75	3	6.25	96.2	80.9
		+	15	1			
	Lower Tx	–	48	0	0	100	100
		+	0	0			
+	Upper Tx	–	1	2	42.9	33.3	40.0
		+	4	3			
	Mid Tx	–	16	2	61.5	88.9	77.4
		+	5	8			
	Lower Tx	–	16	1	0	94.1	76.2
		+	4	0			

*LN* lymph node, *Tx*: Thorax

## Discussion

Our study sought to summarize the characteristics of lymph node metastasis in thoracic T1 sESCC. We found that patients with their tumor invading the layer of proper mucosal (M2) never had lymph node metastasis. The prevalence of lymph node involvement increased with the depth of tumor invasion. Upper mediastinal and (or) abdominal perigastric lymph nodes were most likely involved nodes wherever the tumor was located and whichever layer of submucosa the tumor invaded. Tumors located at lower thoracic segment never had separate cervical lymph node metastasis. The tumor location, lymphatic invasion, and upper mediastinal lymph node metastasis were independent correlated factors with cervical lymph node involvement. The negative predictive value of upper mediastinal lymph node involvement for cervical lymph node metastasis was high for middle and especially lower thoracic sESCC regardless of the status of lymphatic invasion.

Esophagectomy plus lymph node dissection remains the standard treatment especially in more advanced cases. However, there is controversy between surgeons and endoscopists as to which is the optimal option for those tumors invading M2, M3 or SM1. Endoscopic mucosal resection (EMR) and endoscopic submucosal dissection (ESD) are increasingly used to treat superficial esophageal cancer. Such endoscopic procedure could have achieved good survival outcomes had the lymph nodes not been involved. Our results showed that none of the patients with M2 tumor had lymph node metastasis, which was consistent with the literatures [8–10]. These results supported the role of endoscopic procedure as a radical therapy for M2 lesion. Eguchi and colleagues [11] concluded that M1 and M2 lesions were the best candidates for EMR although they demonstrated that the rate of lymph node metastasis in M2 lesion was 5.6%. Eguchi and colleagues [11] showed the lymph node metastatic rate for M3 cancer without lymphatic invasion, M3 cancer with lymphatic invasion, SM1 cancer and SM2/3 cancer was 10.3, 41.7, 53.1 and 53.9% respectively. Araki and his colleagues [3] reported the lymph node metastatic rate for SM1-SM3 was 12.2, 18.4 and 28.6% respectively. A much higher metastatic rate of 24.0, 20.5, 43.8% for SM1, SM2, and SM3 was reported by Li and his colleagues [5]. From the perspective of lymph node metastasis, endoscopic treatment is not suitable for submucosa esophageal cancer. Generally speaking, absolute indication for EMR was restricted to M1 or M2 cancers [12], which is consistent to our results in Table 2.

The incidence of lymph node metastasis and number of positive lymph node increased from SM1 to SM2 to SM3 [3, 13, 14]. According to the literature, the rate of lymph node metastasis in SM3 patients ranged from 25 to 75%, which is similar to progressive esophageal cancer [3, 5, 15, 16]. Surgery is necessary to SM3 patients. Esophagectomy with lymphadenectomy should be adopted for SM2 or SM3 patients. Li and his colleagues [5] reported only one SM3 patient with tumor located at upper third thoracic segment esophagus developed cervical nodal metastases among 20 submucosal patients who underwent three-field lymphadenectomy. In our study, only one patient with lower thoracic tumor had neck lymph node metastasis and the separate neck lymph node metastasis was rare in middle and lower esophageal cancer. Given the low prevalence of cervical metastasis and the high hospital morbidity after 3-field lymphadenectomy, can we avoid the neck surgical incision in selected patients? In our study, tumor location (*p* = 0.006), lymphatic invasion (*p* = 0.001), and upper mediastinal lymph node metastasis (*p* = 0.03) were independent correlated factors with cervical lymph node involvement while no association was found between cervical lymph node metastasis and T stage. This may be attributed to the facts that the subgroups of superficial esophageal cancer were too detailed. Tumor location (*p* = 0.006) is the most significant predictive factor regardless of stage. Tumors located at lower thorax never had separate cervical lymph node metastasis. So the cervical lymph node lymphadenectomy may be unnecessary for these patients if no lymph node metastasis were identified in upper mediastinal lymph node. Lymphatic invasion (*p* = 0.001) is another significant factor for predicting cervical lymph node metastasis. In recent years superficial esophageal cancers with increasing number are removed through endoscopic procedures. The status of lymphatic invasion could be demonstrated through the pathological examination of endoscopicly removed specimen. Therefore, lymphatic invasion is of particular significance in today’s clinical practice.

Sentinel lymph node biopsy (SLNB) was an effective method to detect lymph node metastasis and save surgery time and hospital cost in superficial esophageal cancer [17, 18]. The detection rate and accuracy has been validated by a meta-analysis in early esophageal cancer [19]. However, some disadvantages of SLNB could not be ignored. First, the procedures of SLNB is invasive, which may increase the patient’s preoperative trauma [17]. Moreover, many of the chest lymph nodes are black, which is difficult to distinguish from dyeing agent. The rapid transit of blue dye through lymphatic chain limits its use during the operation time [20]. The prognosis will be poor in patients with negative sentinel lymph node and positive non-sentinel lymph node [17]. In our study, the positive predictive value of upper mediastinal lymph node metastasis was not satisfactory no matter in low-risk or in high-risk patients. However, the negative predictive value was not low, especially for low-risk patients without lymphatic invasion, the negative predictive value of upper mediastinal lymph node metastasis for cervical lymph node metastasis was extremely high (93.8–100%). Even in high-risk patients with lymphatic invasion, the negative predictive value of upper mediastinal lymph node metastasis for cervical lymph node metastasis was also relative high (88.9–84.1%) in middle and lower thoracic tumors patients. This means cervical lymphadenectomy could be avoided for low-risk patients with negative upper mediastinal lymph nodes, particularly for patients with lower thoracic tumors. So, the lymph node metastasis in upper mediastinum can be used as “negativity sentinel lymph node” for negative cervical lymph nodes.

In our study, we excluded patients receiving neoadjuvant therapy, which make it difficult to assess lymphatic invasion. Due to constraints of time and resources, we only made qualitative analyses on lymphatic invasion in the current study. Moreover, this was a retrospective study for superficial ESCC from a single institute, the selective bias and heterogeneity of the patients could not be ignored— the conclusion of our research should be applied with caution. Additionally, the optimal therapeutic method was discussed based on the status of lymph nodes from a surgeon’s point of view. Further clinical trials regarding the prognosis are necessary to establish the best treatment for superficial ESCC.

## Conclusions

In conclusion, tumors invading till proper mucosa were the best indication of EMR. For those without lymphatic invasion, it could be reasonable to avoid cervical lymphadenectomy, if nodes of the upper mediastinum resulted negative at the frozen section, particularly, for those tumors located at lower thorax.

## Data Availability

The datasets used are available from the corresponding author on reasonable request.

## Abbreviations

sESCC: Superficial esophageal squamous cell carcinoma
H.E: Haematoxylin and eosin
EMR: Endoscopic mucosal resection
ESD: Endoscopic submucosal dissection
SLNB: Sentinel lymph node biopsy

## References

[1] Shimizu Y, Kato M, Yamamoto J (2004). EMR combined with chemoradiotherapy: a novel treatment for superficial esophageal squamous-cell carcinoma. Gastrointest Endosc.

[2] Rice TW, Ishwaran H, Hofstetter WL (2016). Recommendations for pathologic staging (pTNM) of cancer of the esophagus and esophagogastric junction for the 8th edition AJCC/UICC staging manuals. Dis Esophagus.

[3] Araki K, Ohno S, Egashira A (2002). Pathologic features of superficial esophageal squamous cell carcinoma with lymph node and distal metastasis. Cancer.

[4] Zhou Y, Du J, Li H (2016). Clinicopathologic analysis of lymph node status in superficial esophageal squamous carcinoma. World J Surg Oncol.

[5] Li B, Chen H, Xiang J (2013). Prevalence of lymph node metastases in superficial esophageal squamous cell carcinoma. J Thorac Cardiovasc Surg.

[6] Davison JM, Landau MS, Luketich JD (2016). A model based on pathologic features of superficial esophageal adenocarcinoma complements clinical node staging in determining risk of metastasis to lymph nodes. Clin Gastroenterol Hepatol.

[7] Wang S, Chen X, Fan J, Lu L (2016). Prognostic significance of Lymphovascular invasion for thoracic esophageal squamous cell carcinoma. Ann Surg Oncol.

[8] Endo M, Yoshino K, Kawano T (2000). Clinicopathologic analysis of lymph node metastasis in surgically resected superficial cancer of the thoracic esophagus. Dis Esophagus.

[9] Liu L, Hofstetter WL, Rashid A (2005). Significance of the depth of tumor invasion and lymph node metastasis in superficially invasive (T1) esophageal adenocarcinoma. Am J Surg Pathol.

[10] Leers JM, DeMeester SR, Oezcelik A (2011). The prevalence of lymph node metastases in patients with T1 esophageal adenocarcinoma a retrospective review of esophagectomy specimens. Ann Surg.

[11] Eguchi T, Nakanishi Y, Shimoda T (2006). Histopathological criteria for additional treatment after endoscopic mucosal resection for esophageal cancer: analysis of 464 surgically resected cases. Mod Pathol.

[12] Higuchi K, Tanabe S, Koizumi W (2007). Expansion of the indications for endoscopic mucosal resection in patients with superficial esophageal carcinoma. Endoscopy.

[13] Ancona E, Rampado S, Cassaro M (2008). Prediction of lymph node status in superficial esophageal carcinoma. Ann Surg Oncol.

[14] Noguchi H, Naomoto Y, Kondo H (2000). Evaluation of endoscopic mucosal resection for superficial esophageal carcinoma. Surg Laparosc Endosc Percutan Tech.

[15] Choi JY, Park YS, Jung HY (2011). Feasibility of endoscopic resection in superficial esophageal squamous carcinoma. Gastrointest Endosc.

[16] Tachibana M, Yoshimura H, Kinugasa S (1997). Clinicopathological features of superficial squamous cell carcinoma of the esophagus. Am J Surg.

[17] Takeuchi H, Kawakubo H, Nakamura R (2015). Clinical significance of sentinel node positivity in patients with superficial esophageal Cancer. World J Surg.

[18] Yuasa Y, Seike J, Yoshida T (2012). Sentinel lymph node biopsy using intraoperative indocyanine green fluorescence imaging navigated with preoperative CT lymphography for superficial esophageal cancer. Ann Surg Oncol.

[19] Filip B, Scarpa M, Cavallin F (2014). Minimally invasive surgery for esophageal cancer: a review on sentinel node concept. Surg Endosc.

[20] Hayashi H, Tangoku A, Suga K (2006). CT lymphography-navigated sentinel lymph node biopsy in patients with superficial esophageal cancer. Surgery.

